# Methionine Restriction Improves Gut Barrier Function by Reshaping Diurnal Rhythms of Inflammation-Related Microbes in Aged Mice

**DOI:** 10.3389/fnut.2021.746592

**Published:** 2021-12-23

**Authors:** Bo Ren, Luanfeng Wang, Aiziguli Mulati, Yan Liu, Zhigang Liu, Xuebo Liu

**Affiliations:** ^1^Laboratory of Functional Chemistry and Nutrition of Food, College of Food Science and Engineering, Northwest A&F University, Yangling, China; ^2^School of Food Science and Pharmaceutical Engineering, Nanjing Normal University, Nanjing, China

**Keywords:** methionine restriction, aging, microbiome diurnal rhythmicity, gut barrier, inflammation

## Abstract

Age-related gut barrier dysfunction and dysbiosis of the gut microbiome play crucial roles in human aging. Dietary methionine restriction (MR) has been reported to extend lifespan and reduce the inflammatory response; however, its protective effects on age-related gut barrier dysfunction remain unclear. Accordingly, we focus on the effects of MR on inflammation and gut function. We found a 3-month methionine-restriction reduced inflammatory factors in the serum of aged mice. Moreover, MR reduced gut permeability in aged mice and increased the levels of the tight junction proteins mRNAs, including those of *occludin, claudin-1*, and *zona occludens-1*. MR significantly reduced bacterial endotoxin lipopolysaccharide concentration in aged mice serum. By using 16s rRNA sequencing to analyze microbiome diurnal rhythmicity during 24 h, we found MR moderately recovered the cyclical fluctuations of the gut microbiome which was disrupted in aged mice, leading to time-specific enhancement of the abundance of short-chain fatty acid-producing and lifespan-promoting microbes. Moreover, MR dampened the oscillation of inflammation-related *TM7-3* and *Staphylococcaceae*. In conclusion, the effects of MR on the gut barrier were likely related to alleviation of the oscillations of inflammation-related microbes. MR can enable nutritional intervention against age-related gut barrier dysfunction.

## Introduction

Aging is accompanied by a decline in the functional capacity of body systems, including cognitive, cardiovascular, and physiological health. The gut has been reported to play crucial roles in the aging process (1). Moreover, aging can alter microbial abundance and cause microbiota dysbiosis (2). Loss of gut microbiota diversity can increase chronic low-grade inflammation and reduce cognitive function during aging (3). The gut microbes could produce short-chain fatty acids (SCFAs) from carbohydrate fermentation. It has been reported that *Sutterella, Bacteroides, Lactobacillus, Prevotella* and *Bacteroidales* are SCFAs producing microbe (4). Moreover, *Desulfovibrionaceae, Staphylococcus*, and *Ruminococcaceae* lipopolysaccharidesare has been reported to be associated with the microbiota inflammatory properties (5, 6). Several studies have revealed that elderly adults and aged mice have lower levels of *Firmicutes, Actinobacteria*, and SCFA-producing microbiota (e.g., *Lachnospiraceae, Faecalibaculum*, and *Ruminococcaceae*) and higher levels of inflammatory gastrointestinal bacteria, such as *Proteobacteria, Desulfovibrio*, and *Staphylococcus*, compared with the microbiome of younger adults and mice (7–10). Age-related changes in the gut microbiome are strongly related to intestinal barrier permeability and age-associated inflammation of the host. *Christensenella, Akkermansia*, and *Bifidobacterium*, showed potential life span–promoting effects (11–13). Furthermore, *Escherichia coli* can induce cognitive impairment and colitis, which is increased in the feces of aged mice and elderly adults (14).

Mammalian circadian clock system is composed of suprachiasmatic nucleus (SCN) in the hypothalamus, which acts as the central pacemaker, and other cells/tissues, such as the liver, gut, and muscle, which act as peripheral oscillators (15, 16). Aging has a negative effect on the circadian clock, and animals and humans show impairment of rest-activity rhythms with age. SCN neurons can disrupt the circadian phase, and the amplitude is reduced in peripheral oscillators with age (17, 18). Mutation of circadian clock gene reduces lifespan and increases oxidative damage and neuronal degeneration in *Drosophila* (19). In addition, disorders of the circadian clock system affect microbial community gene expression, decrease host immune ability and increase the synthesis and transportation of lipopolysaccharide (LPS); these changes are similar to those observed in the gut microbiome of Alzheimer's disease patients (20, 21). Interestingly, the circadian clock is involved in gut microbiota-gut epithelium crosstalk and regulates multiple functions, including intestinal permeability, body composition, and the immune response (22–24). Notably, the abundance of gut bacteria exhibit daily oscillations over a 24-h period (25, 26). The total biomass of the gut microbiome also shows diurnal fluctuations, affecting more than 20% of functional pathways (27). Furthermore, the oscillating pattern of genes in germ-free and wild-type mice was quite different, suggesting that the gut microbiome is associated with host molecular rhythms (28). Similarly, germfree or antibiotic-treated mice exhibit reduced expression of clock and metabolic genes and significantly dampened diurnal histone signaling owing to a lack of histone deacetylase 3 expression (22, 29). The diurnal rhythms of the gut microbiome may contribute to the development of healthy host states. However, whether the circadian rhythm affects the aging process by regulating the gut microbiome remains unclear.

Dietary restriction (DR) extends lifespan in various organisms, including rat, mouse, and fruit fly models, and prevents age-related circadian reprograming in various tissues (30, 31). Methionine is a sulfur-containing essential amino acid that is enriched in animal products. Methionine restriction (MR) can mimic the effects of DR and is effective for suppressing proliferation, increasing longevity, and alleviating inflammation and obesity (32). More specifically, both 80 and 40% MR diets reduce mitochondrial reactive oxygen species generation, which may lead to a healthy lifespan in rodent models (32). In MR-treated mice, the inflammatory responses in the liver and white adipose tissue were found to be significantly downregulated (33). Notably, MR (0.172%) decreases endogenous oxidative molecular damage in the rat brain, further ameliorating aging-related neurodegenerative diseases (34). In addition, a recent study showed that MR improves gut function by regulating the gut microbiome and reducing intestinal permeability in high-fat diet (HFD)-fed mice (35), and feeding of an MR diet for 1 month leads to sex-specific changes in the gut microbiome (36). Moreover, MR time-specifically increases the abundance of SCFA-producing bacteria and decreases the inflammation-related bacteria *Desulfovibrionales* and *Staphylococcaceae* (37), and restriction of sulfur amino acids improves gut barrier function and upregulates claudins (38). Taken together, these findings support that MR may alter the gut microbiome in aged mice. However, whether MR affects diurnal fluctuations in the gut microbiome and the potential effects of MR on age-related gut homeostasis remain unknown. Accordingly, in this study, we revealed the effects of MR on age-related inflammation and gut barrier damages.

## Materials and Methods

### Animal Procedures

Fifteen-month-old and two-month-old male C57BL/6J mice (Vital River Laboratory Animal Technology, Beijing, China) were housed under a 12/12-h light-dark cycle (lights on 08:00, lights off 20:00). Fifteen-month-old mice were randomized into two groups after adaptively feeding. Aged group fed a standard chow diet and MR group fed a MR diet for 3 months. Two-month-old mice were fed a standard chow diet. The detailed dietary compositions of these diets are provided in Supplementary Materials. We have obeyed relevant ethical regulations and research were approved in advance by Northwest A&F University (approval no. N81803231).

### Quantitative Real-Time Polymerase Chain Reaction Analysis

Total RNA was isolated from frozen colon using TRIzol method, diluted to uniform concentration, and reverse-transcribed into cDNA. RT-qPCR was performed using an UltraSYBR Mixture (Cowin Bio., Jiangsu, China) on a CFX96TM real-time system (Bio-Rad, CA, USA), operated at 95°C for 10 min, followed by 40 cycles of 95°C for 15 s and 60°C for 60 s. The 2^−ΔΔCT^ method was used to calculated the mRNA expression. Gene-specific mouse primers were used as listed in Supplementary Materials.

### Serum Lipopolysaccharide Analysis

Serum samples were obtained by eyeball extirpating under anesthesia. Serum LPS contents were detected by ELISA kits (Xinle Bio., Shanghai, China).

### Hematoxylin and Eosin (H&E) and Immunofluorescence Staining

The proximal colons were fixed in paraformaldehyde (4% in PBS, v/v) and embedded in paraffin. For H&E staining, colon sections were cut and stained with hematoxylin and eosin. Three animals per group were used for the assessment of goblet cell numbers. The number of goblet cells on each villus was counted.

For immunofluorescence staining, sections were exposed to Claudin-1 antibodies (Abcam, Ab15098, USA) at 4°C for 10 h, and incubated with biotinylated antibodies. After DAPI staining, the sections were sealed with antifading mounting medium (Solarbio, Beijing, China). Stained sections were observed using an inverted fluorescence microscope (Olympus, Tokyo, Japan).

### Ussing Chamber Assays

Ussing chamber assays were performed as previously described (39). Briefly, 1.5 cm colons were unfolded and placed on Ussing chambers (KingTech, Beijing, China). The chamber was separated into two parts by the unfolded tissue. The tissue was exposed to carbogen-gassed Krebs buffers (7.35 g CaCl_2_·2H_2_O, 13.67 g NaCl, 7.01 g KCl, 4.2 g NaHCO_3_, 4.88 g MgCl_2_·6H_2_O, 3.96 g glucose, 3.74 g NaH_2_PO_4_·2H_2_O, dissolved in 2 L ddH_2_O, pH 7.4) at 37°C. For permeability measurements, fluorescein (0.09 g/L) were replaced on the luminal side and the fluorescence intensity of the serosal buffer was determined on the other side.

### 16S rRNA Sequencing

Fecal samples were collected every 4 h over 24 h before sacrifice through repeated sampling from the same mice after dietary intervention. Total DNA was isolated using an E.Z.N.A. Stool DNA Extraction Kit (Omega, GA, USA), and the V3 to V4 regions of the 16S rRNA gene were amplified using region-specific primers (341_F: 5′-CCTACGGGNGGCWGCAG-3′; and 802_R: 5′-TACNVGGGTATCTAATCC-3′). Raw reads were merged, trimmed, and denoised to construct operational taxonomic units (OTUs) and identified using Usearch (version 7.1). Venn diagram evaluation, β-diversity assessment, principal coordinate analysis (PCoA), and partial least squares-discriminant analysis (PLS-DA) were performed using Qiime2 (2018.11) combined with the R-package vegan and ANCOM2. Taxonomy-based analyses were used to identify significant differences in phylotypes under MR treatment at distinct energy densities. Biomarker-discovery responses to MR were analyzed by linear discriminant analysis (LDA) effect size (LEfSe). Significant biomarkers were detected using the default threshold of α value < 0.05 and LDA score >2.

### Data Analysis

Gut microbiome data were reported as mean ± SEM, other data were reported as max, min, and median. Significant differences between mean values were determined by one-way ANOVA. For multiple comparisons, Tukey's test was performed using GraphPad Prism 7.0 software. Means were considered significantly different when the *p*-value was <0.05. JTK_CYCLE (v3.1) was used to determine whether an OTU was cyclical (ADJ. *p* < 0.05) (40).

## Results

### Effects of MR on Serum Inflammation Factors in Aged Mice

**Figure 1:**
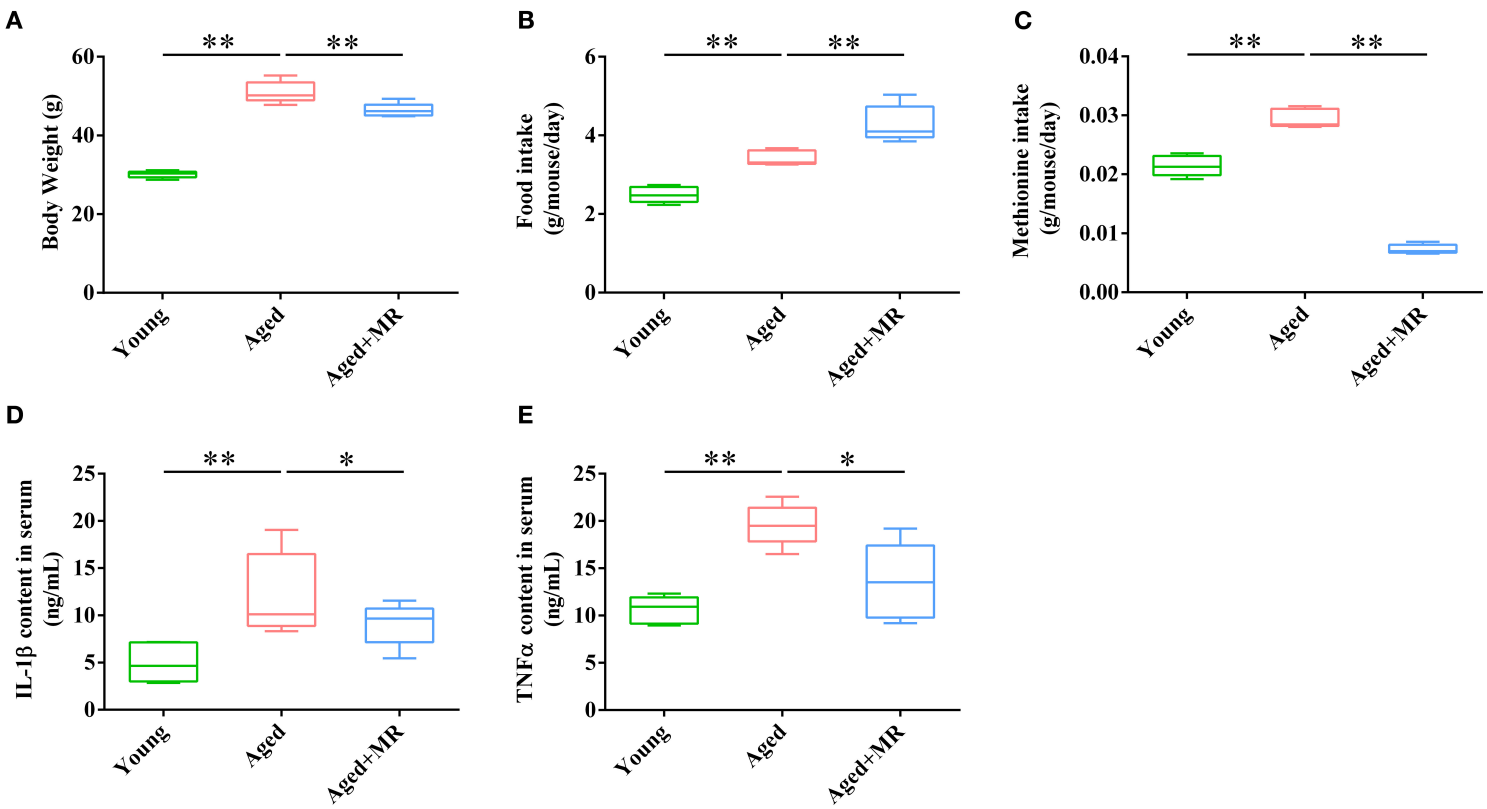
Effects of methionine restriction on the body weight, food intake, and serum cytokines concentrations of aged mice. **(A)** Body weight; **(B)** food intake; **(C)** methionine intake; **(D)** IL-1β content in serum; **(E)** TNF-α content in serum. The boxplot elements are defined as following: center line, median; box limits, upper and lower quartiles; whiskers, min to max (*n* = 5/group). Significant differences between mean values were determined using one-way ANOVA analysis of variance with Tukey's multiple comparison test; *p < 0.05, **p < 0.01.

Aged mice were fed with an MR diet for 3 months. MR feeding significantly decreased the body weights of aged mice (*p* < 0.01, Figure 1A). However, MR-fed aged mice consumed more food compared to control diet-fed aged mice (*p* < 0.01, Figure 1B). Moreover, methionine intake was significantly decreased under MR feeding (*p* < 0.01, Figure 1C). The inflammatory response during aging is one of the causes of other age-related symptoms. To evaluate the effects of MR on age-induced systemic inflammation, which have been reported to be a risk factor for age-related biological degradation, inflammatory factors were investigated. As shown in Figures 1D,E, interleukin (IL)-1β and tumor necrosis factor (TNF)-α levels in serum were dramatically increased in aged mice, and this effect was reversed in MR-fed aged mice (Figures 1D,E, *p* < 0.05).

### Effects of MR on the Gut Barrier Integrity in Aged Mice

**Figure 2:**
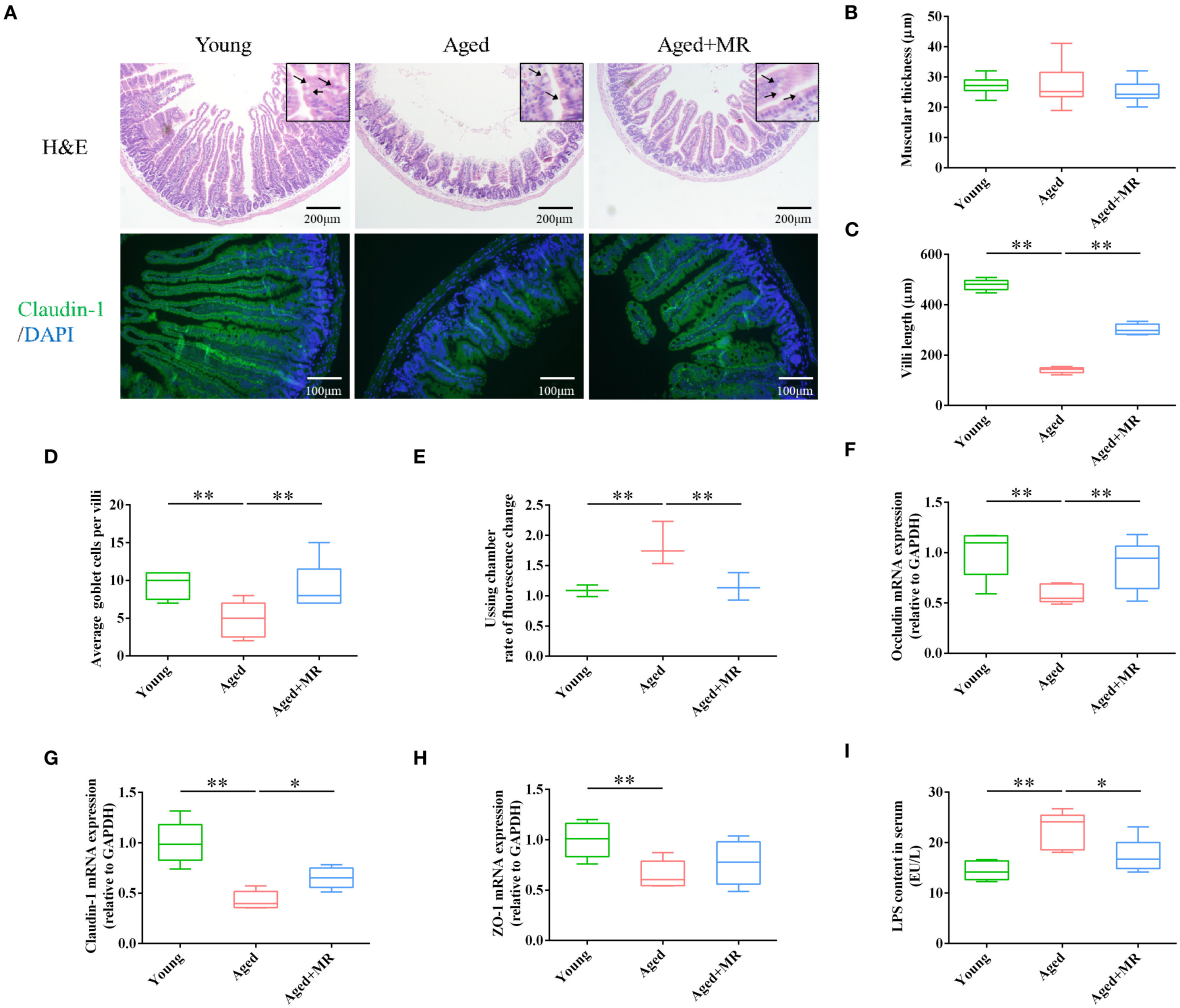
Effects of methionine restriction on the gut barrier integrity of aged mice. **(A)** Representative images of hematoxylin and eosin staining and immunofluorescence staining of claudin-1/DAPI in mouse colon tissues (*n* = 3/group). Arrows point to goblet cells. **(B)** Muscular thickness; **(C)** villus length; **(D)** average goblet cell per villi; **(E)** rate of fluorescence changes during Ussing chamber analysis (*n* = 3/group). **(F–H)** mRNA expression of *occludin, claudin-1*, and *Zo-1*; loading control: *Gapdh* (*n* = 5/group). **(I)** LPS content in serum. (*n* = 5/group). The boxplot elements are defined as following: center line, median; box limits, upper and lower quartiles; whiskers, min to max. Significant differences between mean values were determined using one-way ANOVA analysis of variance with Tukey's multiple comparison test; *p < 0.05, **p < 0.01.

Intestinal barrier integrity plays a critical role in age-related systemic inflammation. There is no significant difference of muscular thickness between young and aged mice. We found MR increased the villi length, and number of goblet cells in aged mice (*p* < 0.01, Figures 2A–D). Moreover, immunofluorescence staining for claudin-1 showed that aging decreased the expression of claudin-1, whereas MR increased claudin-1 in aged mice (Figure 2A). Furthermore, we observed that MR prevented gut leakage (Figure 2E). In addition, the expression levels of occludin, claudin-1, and zona occuldens-1 (*Zo-1*) mRNAs, which encode tight-junction proteins in the gut barrier, were also elevated by MR (*p* < 0.01, Figures 2F–H). The intestinal barrier is the first defense against LPS-induced inflammatory activation in the body. Consistent with this, LPS content was increased in aged mouse serum, whereas MR reversed the abnormal increase (*p* < 0.05, Figure 2I). This result showed that MR reduced age-related inflammation, possibly because of its effects on improving gut barrier function.

### Effects of MR on Gut Microbiome in Aged Mice

**Figure 3:**
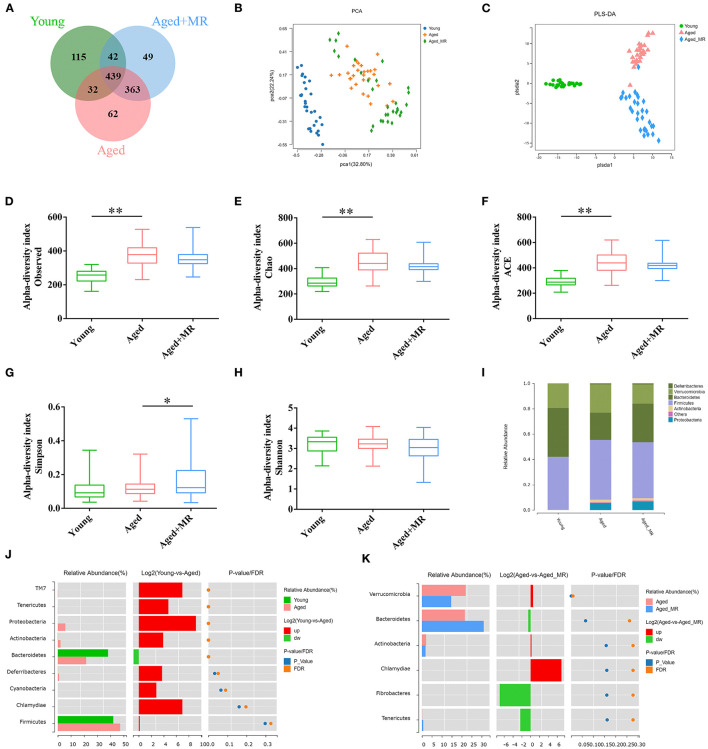
Effects of methionine restriction on the gut microbiota composition of aged mice. **(A)** Venn diagram displays the number of shared and unique OTUs among Young, Aged and Aged+MR. **(B)** Principal coordinate analysis (PCoA) and **(C)** Partial least squares discrimination analysis (PLS-DA) indicated the beta-diversity of gut microbiome.; **(D)** Observed species index, **(E)** Chao index, **(F)** Ace index, **(G)** Simpson index, and **(H)** Shannon index indicated the alpha-diversity of gut microbiome. The boxplot elements are defined as following: center line, median; box limits, upper and lower quartiles; whiskers, min to max. Significant differences between mean values were determined using one-way ANOVA analysis of variance with Tukey's multiple comparison test; *p < 0.05, **p < 0.01. **(I)** Gut microbiome composition at the phylum level. Differential analysis of the gut microbiome between **(J)** young and **(K)** aged + MR groups.

We identified 1102 OTUs in mice fecal of 3 conditions. Venn diagram showed that microbial composition of mice in each group was significantly changed (Figure 3A). As shown in Figures 3B,C, the gut microbiome was distinctly different between the young and aged groups, as demonstrated by PCA and PLS-DA. Moreover, aging significantly increased the observed OTUs, Chao index, and ACE index compared with that in young mice (*p* < 0.01, Figures 3D–G). However, MR increased the Simpson index in aged mice (*p* < 0.01, Figure 3H). MR affected the relative abundance of gut microbiome. Specifically, compared with the young group, the proportion of *Bacteroidetes* decreased and the proportions of *Firmicutes* and *Proteobacteria* increased in the aged group. After MR treatment, an increase in the proportion of *Bacteroidetes* with a decrease in *Firmicutes* was observed (Figure 3I). LEfSe analysis suggested that *Akkermansia, Verrucomicrobiaceae*, and *Bacteroidaceae* were universal markers in the young group; *Erysipelotrichaceae, Staphylococcus*, and *Helicobacteraceae* were dominant microbes in the aged group; and *Prevotella, Bacteroidales, Desulfovibrionales*, and *Bifidobacteriales* were aged + MR-specific markers (Figures 3J,K).

### Differential Effects of MR on the Gut Microbiome Composition Over a 24-H Period

**Figure 4:**
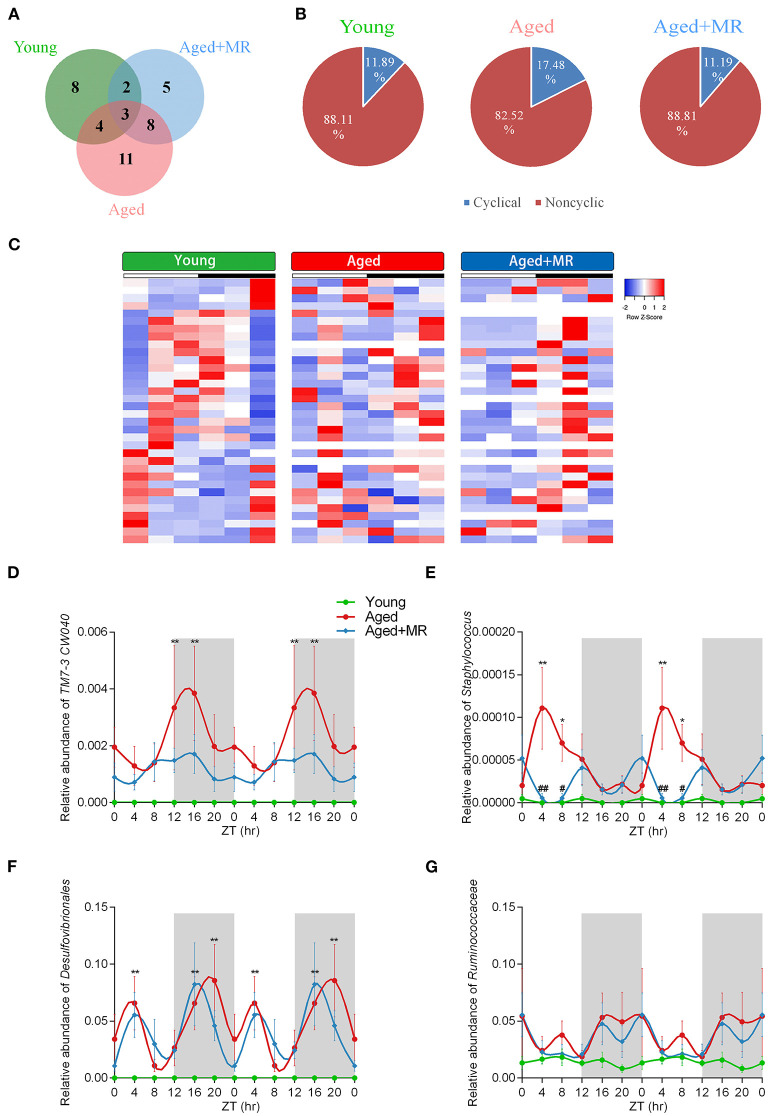
Effects of methionine restriction on diurnal rhythms of the gut microbiome in aged mice. **(A)** Venn diagrams displays the number of shared and unique cycling OTUs. **(B)** Proportion of cycling and non-cycling OTUs. **(C)** Heatmap of cycling OTUs organized by peak, from the young group. **(D–G)** Diurnal patterns of relative abundances of *TM7-3 CW040, Staphylococcus, Desulfovibrionales*, and *Ruminococcaceae* over 24 h. Data are double-plotted for clear visualization. Data are presented as means ± SEMs; *n* = 5/time point. *p < 0.05, **p < 0.01, vs. the young group; ^#^*p* < 0.05, ^##^*p* < 0.01, vs. the aged group. Significant differences between means were determined using one-way ANOVA analysis of variance with Tukey's multiple-comparison test.

To investigate the stability of the relative abundance of gut microbes over the course of a day, we use JTK_CYCLE to define the rhythmicity of each OTU. Venn diagram analysis showed the distribution of OTUs that were considered to be rhythmic, with an ADJ. *p*-value <0.05, in each group (Figure 4A). Moreover, the gut microbiome of aged mice had the most rhythmic OTUs, accounting for 17.48% of the total number of OTUs. However, the gut microbiome of young mice and MR-fed aged mice ranged from 11 to 12% (Figure 4B). In OTUs with oscillations, peaks appeared at different times. In the young group, the cycling OTUs showed peaks distributed over a day (Figure 4C). The peaks of these OTUs changed in the feces of aged mice. However, these alterations were alleviated by MRD. The peaks of these OTUs were shifted rather than dramatically disturbed compared with those in young mice (Figure 4C). These data indicated that the composition of the gut microbiome changed over time and that MR could have different influences on the gut microbiome at different time points.

### MR Decreased the Inflammation-Related Microbiome in Aged Mice

The results of analyses of inflammation-related microbiome, involving *TM7-3 CW040, Staphylococcaceae, Desulfovibrionales*, and *Ruminococcaceae*, showed that the aged group exhibited dramatic increases in *TM7-3 CW040, Staphylococcaceae*, and *Desulfovibrionales* compared with the young mice. No significant differences in *Ruminococcaceae* were detected between the young and aged groups. The relative abundance of *Staphylococcaceae* was lower in the aged + MR group than in the aged group at ZT4 and ZT8. Moreover, the aged group showed disordered cyclical oscillations of *Staphylococcaceae, Desulfovibrionales*, and *Ruminococcaceae* (ADJ. *p* = 0.18, 0.08, and 0.05, respectively). After MR feeding, the aged + MR group exhibited similar cyclical fluctuations in *Staphylococcaceae, Desulfovibrionales*, and *Ruminococcaceae* as the young group (Figures 4D–G).

### MR Increased the SCFA-Producing and Life Span-Promoting Microbiome in Aged Mice

**Figure 5:**
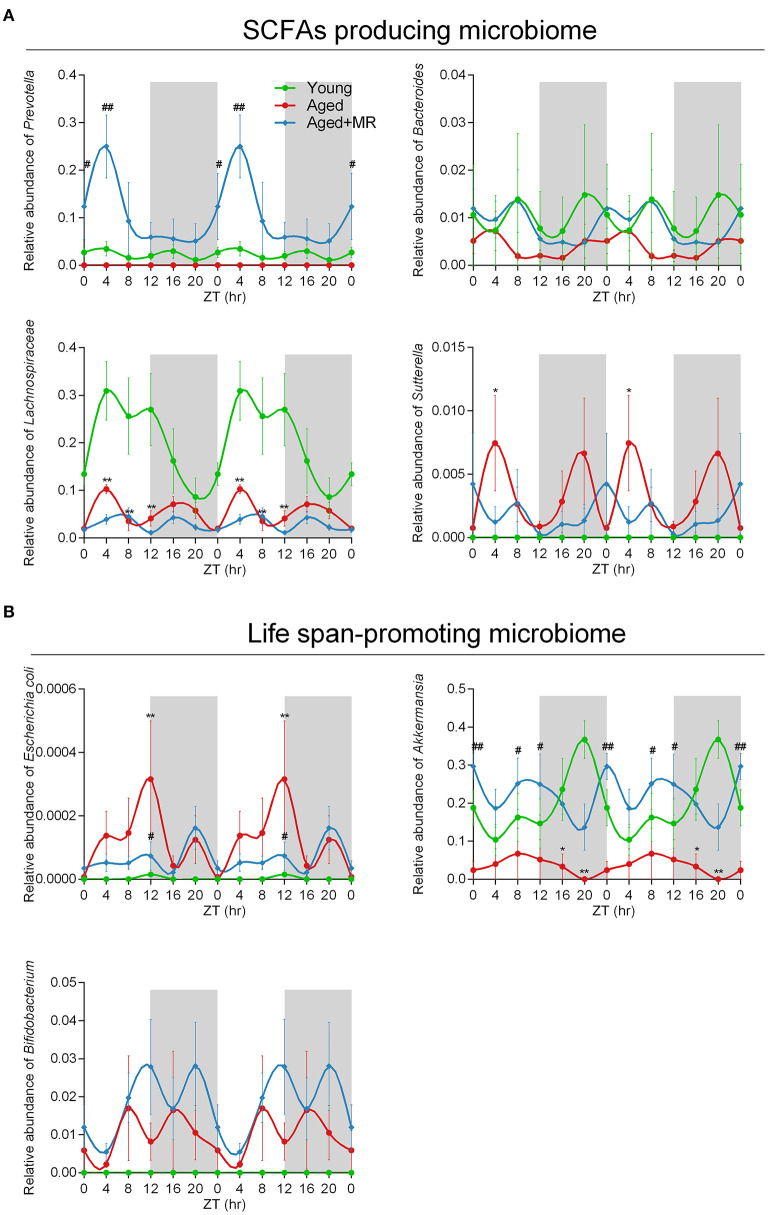
Methionine restriction increased the abundances of SCFA-producing and life span-promoting microbes. **(A)** Diurnal patterns of the relative abundances of *Prevotella, Bacteroides, Lachnospiraceae*, and *Sutterella* over 24 h. **(B)** Diurnal patterns of the relative abundances of *Escherichia coli, Akkermansia*, and *Bifidobacterium* over 24 h. Data are presented as means ± SEMs; *n* = 5/time point. *p < 0.05, **p < 0.01, vs. the young group; ^#^*p* < 0.05, ^##^*p* < 0.01, vs. the aged group. Significant differences between means were determined using one-way ANOVA analysis of variance with Tukey's multiple-comparison test.

To consider circadian effects, the family, genus, and species levels of the gut microbiome were evaluated. Specific differences in the relative abundances of these organisms are shown in Figure 5A, including SCFA-producing microbes (e.g., *Prevotella, Bacteroidales, Bacteroides, Lachnospiraceae*, and *Sutterella*) and potential life span-promoting microbes (e.g., *Escherichia coli, Akkermansia*, and *Bifidobacterium*), from the light to the dark phase. The aged + MR group showed a marked increase in the relative abundance of *Prevotella* compared with the aged group, particularly at ZT0 and ZT4. Moreover, the young group exhibited higher relative abundances of *Bacteroidales, Bacteroides*, and *Lachnospiraceae* than the aged groups, but had a lower relative abundance of *Sutterella*. A previous study demonstrated that aging could alter the *Sutterella* to *Barneseilla* ratio (41). Additionally, we found that MR significantly increased the relative abundance of *Bacteroidales* at ZT0. Although *Lachnospiraceae* has been found to increase SCFA levels, this microbe is also associated with obesity. Our findings showed that MR slightly decreased the relative abundance of *Lachnospiraceae* in the aged mice. In addition, in the young and aged + MR groups, *Prevotella, Bacteroides, Lachnospiraceae, Escherichia coli*, and *Bifidobacterium* exhibited similar cyclical oscillation, whereas the cyclical fluctuations of *Bacteroides* and *Bifidobacterium* were disrupted (ADJ. *p* = 0.17 and 0.08, respectively) compared with those in the young and aged + MR groups (Figure 5B). These results together indicated that MR enhanced the relative abundances of SCFA-producing and life span-promoting microbiomes and restored the diurnal fluctuations of specific microbiomes in aged mice.

### Correlation Analysis Between Characteristic Indicators and the Specific Rhythm Microbiome

**Figure 6:**
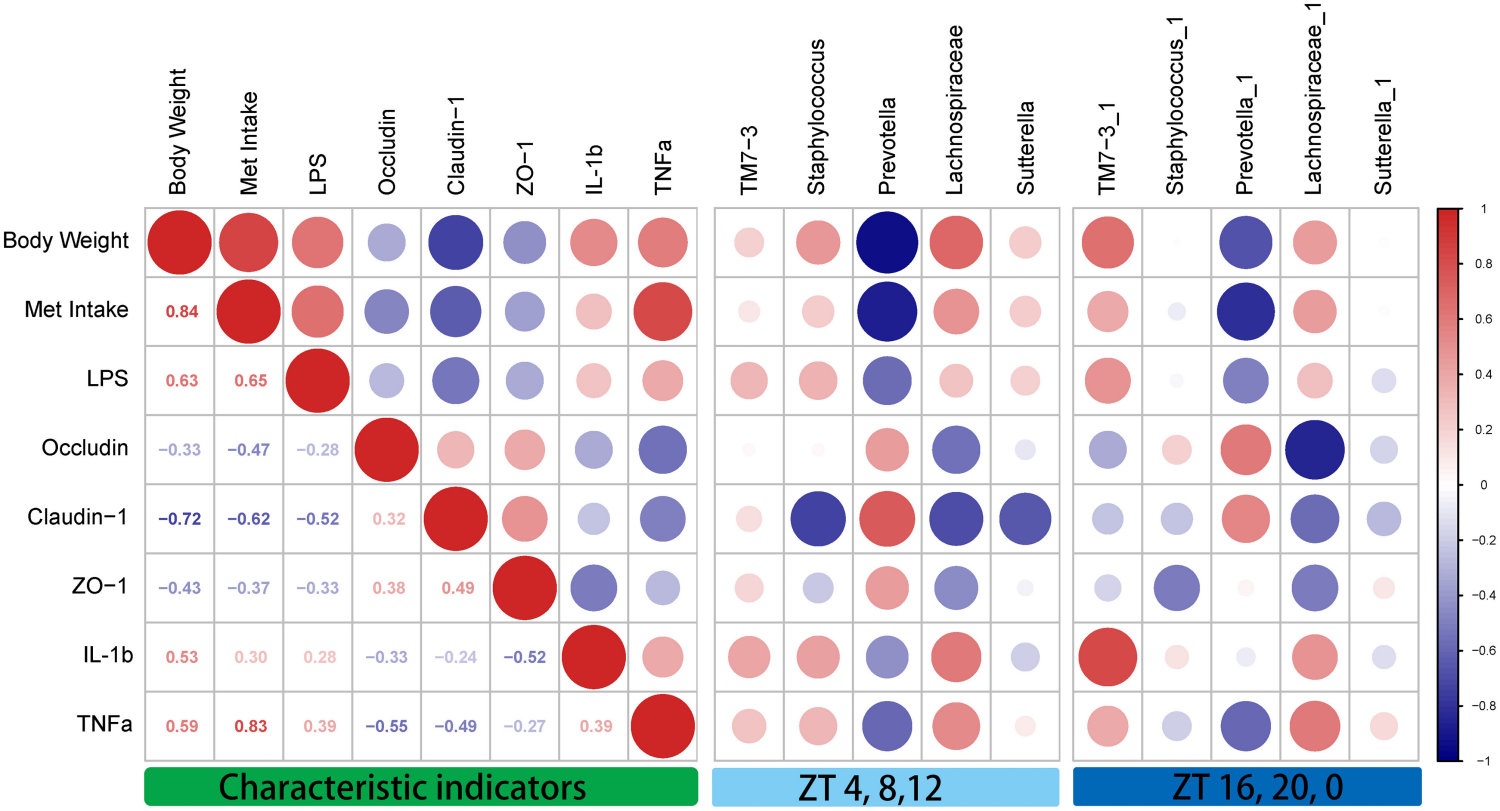
Correlation analysis between the characteristic indicators and relative abundances of the gut microbiome during daytime (ZT4, ZT8, and ZT12) and nighttime (ZT16, ZT20, and ZT0). Pearson's correlation analysis was performed for body weight, methionine intake, inflammatory factors, and relative abundance of the gut microbiome. Red, positive correlation; blue, negative correlation.

To clarify the correlations among the characteristic indicators, tight junction proteins, and inflammatory factors, Pearson's correlation analysis was performed (Figure 6). The results showed that characteristic indicators and specific rhythm microbiomes were highly correlated. More specifically, Met intake was positively correlated with serum TNF-α levels (Pearson's coefficient = 0.83). Importantly, the correlation of oscillation characteristic indicators and abundance in the light period (ZT4, ZT8, and ZT12) was slightly higher than that in the dark period (ZT16, ZT20, and ZT0).

## Discussion

**Figure 7:**
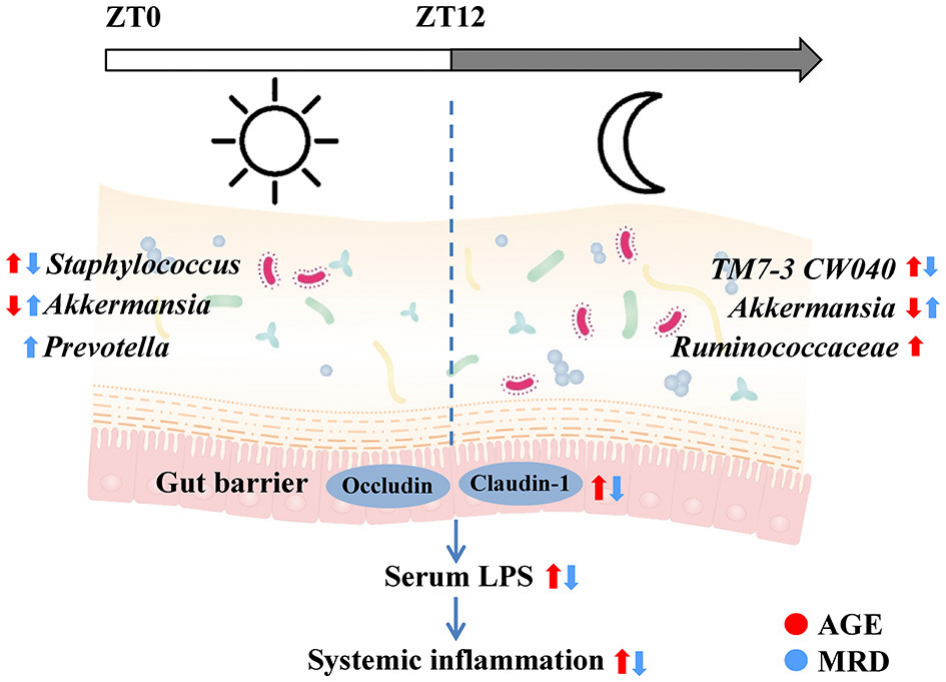
Methionine restriction alleviated systemic inflammation in aged mice by reshaping the gut microbiome, improving gut barrier function, and reducing serum lipopolysaccharide.

In this paper, the protective effects of MR on age-related systemic inflammation were investigated. The mechanism may involve protecting the gut barrier and reducing LPS, consistent with the results of reshaping the gut microbiome (Figure 7). We also found that intestinal tight junction proteins were upregulated and the age-related rhythmic disturbance of intestinal bacteria was alleviated by MR.

The effects of MR on lifespan extension have been reported in various animal models (32). Recent studies have shown that MR can alleviate age-related bone density reduction, cognitive dysfunction, systemic inflammation, and oxidative stress (35, 42–44). Moreover, during aging, intestinal function gradually degrades, which may facilitate other age-related degenerative processes. Aging leads to aging of intestinal cells, damage to intestinal barrier function, and reduction of intestinal tight junction protein levels, which in turn promotes the degradation of overall body function (3, 45, 46). Additionally, improving aging-related intestinal barrier damage can extend lifespan (46, 47). Mechanistically, MR can promote the production of glutathione and regulate the production of H_2_S by activating the nutritional response signal fibroblast growth factor 21, thereby prolonging lifespan and reducing inflammation (48, 49). However, some studies have shown that the anti-inflammatory effects of MR are partly independent of these mechanisms (50). Furthermore, the regulatory effects of MR on the intestinal function of aging mice have not been clearly studied. In an HFD-induced obese mouse model, an MRD reshaped the gut microbiome, reduced inflammatory factors, and ultimately alleviated metabolic syndrome in obese mice (35, 37). MR alleviates fat accumulation and inflammatory responses by reshaping gut microbiome of 8-month-old mice (51). In young mice, dietary methionine supplementation alters one-carbon metabolism and DNA methylation in the proximal jejunum, and alters the normal gut physiology (52). Increasing or decreasing the level of methionine in the diet will cause changes in the composition of gut microbes, with some differences between males and females (36). Consistent with this, in our study, dramatic changes in gut microbiome composition were found in MR-treated 18-month-old mice, with a lower level of inflammatory factors.

Intestinal barrier integrity is necessary for maintaining the stability of the body. Gut microbial disruption leads to compromised gut barrier function and sustained systemic inflammation (53). Microbiome-host interactions are related to the inflammatory factors production in humans (54), and LPS produced by the gut microbiota accelerates inflammation and aging in mice (55). In the current study, we found that MR reduced serum LPS levels in aged mice and that LPS levels were correlated with Met intake. In addition, the increase in LPS associated with age further activates the intestinal inflammatory factors TNF-α and IL-1β, triggering a systemic inflammatory response. In another study in our group, an increase in LPS caused by aging was found to exacerbate the cognitive impairment associated with aging (56). Furthermore, damage to the intestinal barrier function and activation of inflammatory factors have been used as triggers or biomarkers for Crohn's disease, Parkinson's disease, and metabolic syndrome (57–59). We found that MR reduced the levels of serum LPS and inflammatory factors in aged mice, which may be related to enhancement of the integrity of the intestinal barrier and upregulation of tight junction proteins by MR. The results of correlation analysis also supported this speculation.

The gut microbiome has a circadian rhythm similar to that of organisms, and this rhythm plays critical roles in the biological functions of the host, including digestion, absorption, and metabolism (60, 61). Feeding time and food composition have been shown to affect the diurnal rhythm of the gut microbiome (62, 63). In our study, MR affected the diurnal rhythms of some specific intestinal bacteria, particularly those related to inflammation-associated, SCFA-producing, and lifespan-promoting microbes. The relative abundance of *TM-7* has been reported to be increased in the intestine and oral cavity of patients with inflammatory bowel disease (64, 65). In addition, the relative abundances of *Desulfovibrionaceae* and *Staphylococcaceae*, related to the activation of host inflammatory factors, are down-regulated by MR. In particular, *Desulfovibrio* and other sulfate-reducing bacteria, hydrogen sulfide producer, are related to inflammatory response (66). *Sutterella, Bacteroides, Lactobacillus, Prevotella*, and *Bacteroidales* are associated with SCFA production (4). *Akkermansia* improves the colonic mucus thickness decline and attenuates immune activation in aged mice (67). *Bifidobacterium* and *Lactobacillus* have been reported to impair brain function and further lead to neurodegenerative disorders (68). We found that MR increased the relative abundance of *Staphylococcaceae* during the daytime and decrease the relative abundance of *TM7-3 CW040* at night in aged mice. MR has no difference in up-regulating the relative abundance of *Akkermansia* between a day.

This study has potential limitations. Firstly, the synchronism between gut microbiota and gut barrier function upon MR diets would be estimated if there are serum LPS concentrations with different time points. Secondly, there may be commensal bacteria that become opportunistic pathogens only under certain conditions. The classification of inflammation-related, SCFA producing and age-related microbes in presented study may not be applicable to all pathologies. Metagenomics sequencing could provide more precise information on how gut microbiota affects host functions. Thirdly, we only chose a single dose of Met-restricted diet commonly used in MR studies. The effect of different Met intakes on the gut microbiome is not clear. A wider range of Met-restricted level will reveal a clearly correlation between Met intake and the relative abundance of gut bacteria. Moreover, feeding time was not monitored in this study, which was proved to have great influence on the diurnal rhythm of the gut microbiome. The relationship between dietary methionine content and the relative abundance of gut microbiome on aging and aging-related gut dysfunction are under further investigation.

## Conclusion

In this study, we found that MR alleviated systemic inflammation, improved gut barrier function, and reshaped the gut microbiome. The effects of MR on *Staphylococcus, TM7-3 CW040*, and *Prevotella* varied during the 24-h observation period. These findings provide important insights into the application of MR for prevention of age-related gut barrier damage and inflammation-related diseases.

## Data Availability Statement

The datasets presented in this study can be found in online repositories. The names of the repository/repositories and accession number(s) can be found below: https://www.ncbi.nlm.nih.gov/Traces/study/?acc=PRJNA747265.

## Ethics Statement

The animal study was reviewed and approved by Animal Studies Committee at Northwest A&F University.

## Author Contributions

BR, LW, AM, and YL performed the experiments and analyzed the data. ZL and XL designed the study. BR, LW, and ZL wrote the paper. BR, AM, and YL prepared the figures. All authors discussed the results and commented on the paper.

## Funding

This work was financially supported by the National Natural Science Foundation of China (Nos. 32072214 and 81871118).

## Conflict of Interest

The authors declare that the research was conducted in the absence of any commercial or financial relationships that could be construed as a potential conflict of interest.

## Publisher's Note

All claims expressed in this article are solely those of the authors and do not necessarily represent those of their affiliated organizations, or those of the publisher, the editors and the reviewers. Any product that may be evaluated in this article, or claim that may be made by its manufacturer, is not guaranteed or endorsed by the publisher.
