# Supplementary material for: Methionine Restriction Improves Gut Barrier Function by Reshaping Diurnal Rhythms of Inflammation-Related Microbes in Aged Mice
Source: Front Nutr. 2021 Dec 23;8:746592. doi: 10.3389/fnut.2021.746592 (PMC8733897; doi:10.3389/fnut.2021.746592)
Supplement: Supplementary file 1 [file Data_Sheet_1.DOCX]

Supplementary Materials for

**Methionine restriction improves gut barrier function by reshaping diurnal rhythms of inflammation-related microbes in aged mice**

Bo Ren et al.

**This PDF file includes:**

Supplementary Figures 1

Supplementary Tables 1 - 2

**Supplemental Figures**

**
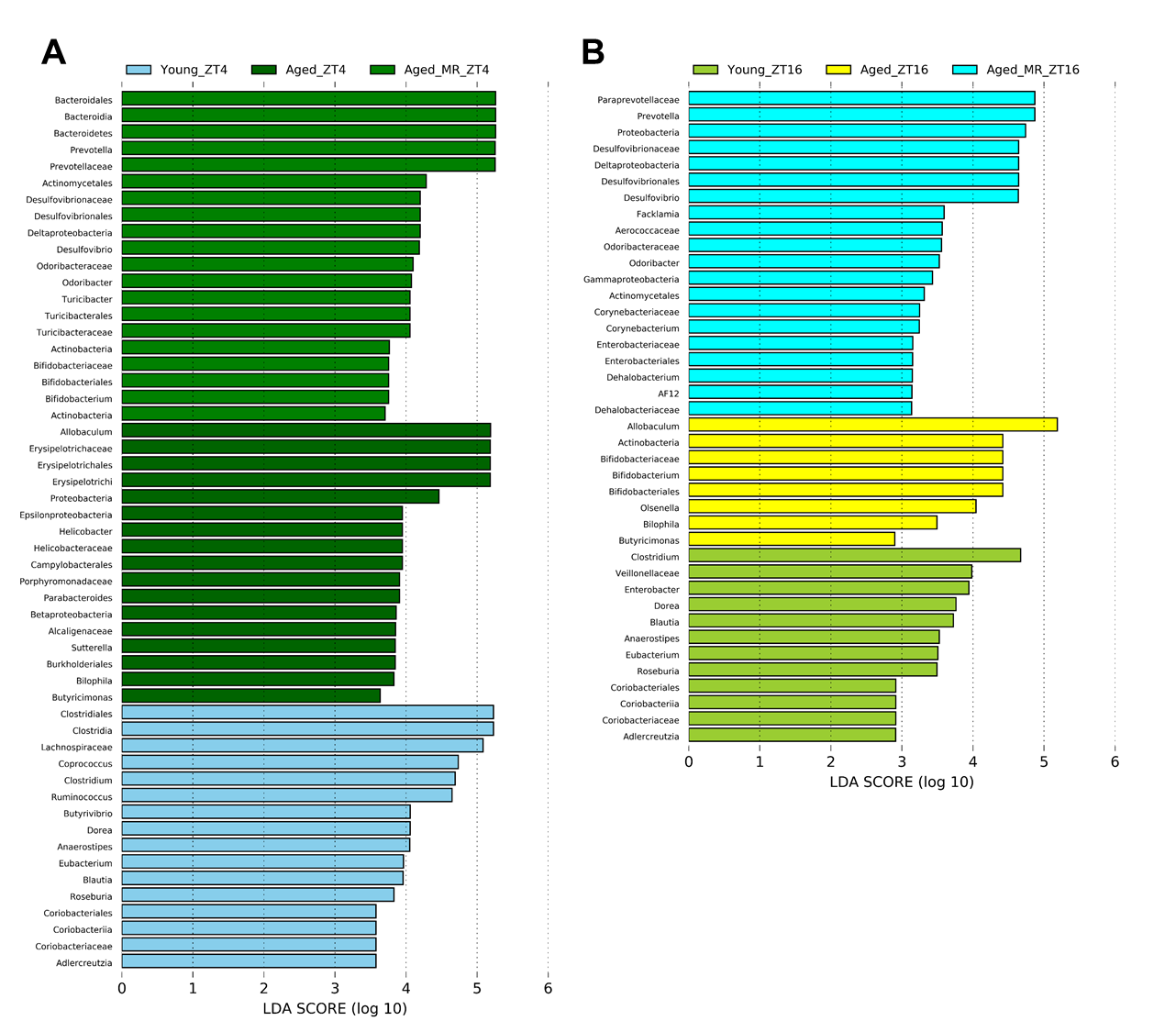
**

**Supplementary Figure 1. Effect of MR on gut microbiome of aging mice at ZT4 and ZT16**

LDA effect size (LEfSe)-based identification of taxa showing maximal differences in relative abundance between Aged and Aged+MR at ZT4 and ZT16 (n = 5 per ZT).

**Supplementary Table 1** The ingredients of the experimental diets (g/1000 g of diet)

| **Diet** | **Control methionine diet (0.86% methionine)** | **Methionine restriction diet (0.17% methionine)** |
| --- | --- | --- |
| Custom mixture of L-amino acids  (no L-methionine, cysteine, and L-glutamic acid) | 102.8 | 102.8 |
| L-methionine | 8.6 | 1.7 |
| L-glutamic acid | 25.9 | 32.8 |
| [Saccharose](javascript:;) | 211.9 | 211.9 |
| Maltodextrin | 53.0 | 53.0 |
| Corn Starch | 458.3 | 458.3 |
| Cellulose | 50.0 | 50.0 |
| Choline Bitartrate | 2.1 | 2.1 |
| Vitamin and Mineral mix | 45 | 45 |
| [CornOil](javascript:;) | 42.4 | 42.4 |
| Lard | 0 | 0 |
| Fat ratio (%) | 10% | 10% |
| L-methionine ratio (%) | 0.86% | 0.17% |

**Supplementary Table 2** The Primer sequences used for RT-qPCR

| **Gene name** | **Forward Primer** | **Reverse Primer** |
| --- | --- | --- |
| *Claudin-1* | CGACTCCAAACACTGGAACTCA | GCCTGCTTCTCATCTGTTGTCA |
| *Occludin* | ATGTCCGGCCGATGCTCTC | TTTGGCTGCTCTTGGGTCTGTAT |
| *Zo-1* | TGGGAACAGCACACAGTGAC | GCTGGCCCTCCTTTTAACAC |
| *Gapdh* | TCACCACCATGGAGAAGGC | GCTAAGCAGTTGGTGGTGCA |
